# Transovarial Transmission of *Anaplasma marginale* in *Rhipicephalus* (*Boophilus*) *microplus* Ticks Results in a Bottleneck for Strain Diversity

**DOI:** 10.3390/pathogens12081010

**Published:** 2023-08-02

**Authors:** Sofía de la Fournière, Eliana Carolina Guillemi, Martina Soledad Paoletta, Agustina Pérez, Dasiel Obregón, Alejandro Cabezas-Cruz, Néstor Fabián Sarmiento, Marisa Diana Farber

**Affiliations:** 1 Instituto de Agrobiotecnología y Biología Molecular (IABIMO) INTA—CONICET, P.O. Box 25, Hurlingham B1686LQF, Argentina; sofidlf@gmail.com (S.d.l.F.); guillemi.eliana@inta.gob.ar (E.C.G.); paoletta.martina@inta.gob.ar (M.S.P.); perez.agustina@inta.gob.ar (A.P.); 2 School of Environmental Sciences, University of Guelph, Guelph, ON N1H 2W1, Canada; dasielogv@gmail.com; 3 Anses, INRAE, Ecole Nationale Vétérinarie d’Alfort, UMR BIPAR, Laboratoire de Santé Animale, F-94700 Maisons-Alfort, France; alejandro.cabezas@vet-alfort.fr; 4 Estación Experimental Agropecuaria Mercedes, Instituto Nacional de Tecnología Agropecuaria, Mercedes 3470, Argentina; sarmiento.nestor@inta.gob.ar

**Keywords:** *Anaplasma marginale*, *Rhipicephalus microplus*, transovarial transmission, vector competence, MSP1a

## Abstract

*Anaplasma marginale* is an obligate intraerythrocytic bacterium of bovines, responsible for large economic losses worldwide. It is mainly transmitted by *Rhipicephalus (Boophilus) microplus* ticks and, despite mounting evidence suggesting transovarial transmission, the occurrence of this phenomenon remains controversial. We evaluated the vector competence of *R. microplus* larvae vertically infected with *A. marginale* to transmit the bacterium to a naïve bovine. A subgroup of engorged female ticks collected from an *A. marginale*-positive animal was dissected and the presence of the pathogen in its tissues was confirmed. A second subgroup of ticks was placed under controlled conditions for oviposition. After confirming the presence of *A. marginale* in the hatched larvae, an experimental infestation assay was conducted. Larvae were placed on an *A. marginale*-free splenectomized calf. The bacterium was detected in the experimentally infested bovine 22 days post-infestation. We analyzed the *A. marginale* diversity throughout the transmission cycle using the molecular marker MSP1a. Different genotypes were detected in the mammalian and arthropod hosts showing a reduction of strain diversity along the transmission process. Our results demonstrate the vertical transmission of *A. marginale* from *R. microplus* females to its larvae, their vector competence to transmit the pathogen, and a bottleneck in *A. marginale* strain diversity.

## 1. Introduction

*Anaplasma marginale* is a gram-negative alpha-proteobacteria from the Anaplasmataceae family in the order Rickettsiales. This obligate intracellular microorganism infects erythrocytes of ruminants, such as cattle, water buffalo, bison, and deer [1,2,3,4,5], but it has also been reported infecting non-ruminant species such as the giant anteater (*Myrmecophaga tridactyla*) [6], dogs [7] and equines [8]. *Anaplasma marginale* is responsible for significant economic losses to the beef and dairy industries worldwide because of lower weight gain rates, lower milk production, abortions, high treatment costs, and mortality [9]. *Anaplasma marginale* transmission can be due to poor management practices, shared use of contaminated medical instruments, or hematophagous arthropods [10]. In this regard, the predominant vectors worldwide are ticks and in particular *Rhipicephalus (Boophilus) microplus* Castrini (Acari: Ixodidae) [11], which are distributed in tropical and subtropical areas of high livestock production [12]. Furthermore, horseflies have been associated with *A. marginale* transmission [13,14]. *A. marginale* establishes persistent infection in cattle with cyclic low-level rickettsemia, thus serving as a reservoir for both tick and mechanical transmission [9]. The transplacental transmission of *A. marginale* has been broadly described both in experimental studies and in field conditions [1].

*Rhipicephalus microplus* is considered to be the most important vector of *A. marginale* in cattle [15]. Taking into consideration that *R. microplus* is a one-host tick (a tick that preferentially completes its life cycle on a single host species) and a low proportion of males may migrate in search of females, the role of males in the biological transmission of *A. marginale* would be of relevance as they can transmit the bacterium repeatedly when transferring among cattle [16]. However, studies suggested that the proportion of adult *R. microplus* males migrating between cattle under natural field conditions is too low to play a role as the main transmission route in the enzootic regions of Argentina [17]. Although *A. marginale* DNA was detected in *R. microplus* larvae according to PCR analyses [18], the potential importance of the transovarial transmission route for the circulation of the bacterium remains neglected [19].

Previous studies evaluating the transovarial transmission of *A. marginale* in *R. microplus* concluded that this phenomenon did not occur under certain experimental conditions [20,21,22,23]. Other authors have shown that the offspring larvae of *R. microplus* females infected with *A. marginale* were positive by PCR even though they did not evaluate whether these larvae were able to transmit the bacterium to calves [18] thus, vector competence [24] remains unstudied for this tick species. Although it is generally accepted that transovarial transmission of *Anaplasma* spp. by tick vectors is either inefficient or nonexistent [25], results similar to those for *A. marginale* were reported for other *Anaplasma* species. For *A. platys*, a recent study demonstrated a highly efficient transovarial transmission by *R. sanguineus* sensu stricto ticks [26], while *A. phagocytophilum* DNA was detected in questing larvae of *Haemaphysalis megaspinosa*, *H. longicornis*, and *Ixodes ricinus*, indicating the possibility for transovarial transmission of the pathogen in these tick species [27,28,29].

In this research, we evaluated the transovarial transmission of *A. marginale* in the *R. microplus* tick collected from the field and studied the vector competence of the infected tick larvae to transmit the transovarially acquired *A. marginale* to a susceptible bovine. We also studied the genotypic diversity of *A. marginale* in the different stages of the transmission cycle.

## 2. Materials and Methods

### 2.1. Ethical Statement

The study was conducted under the guidelines of the Institutional Committee for the Use and Care of Experimentation Animals, CICUAE committee, Corrientes, Argentina (protocol number 02/2018).

### 2.2. Study Design

**Figure 1:**
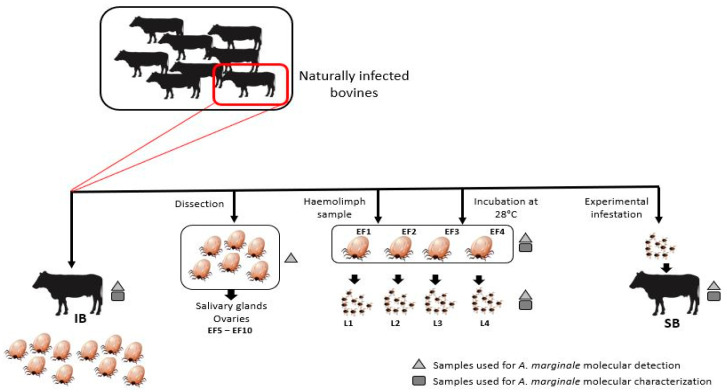
Schematic representation of the experimental design. IB: naturally infected bovine (donor calf), EF: engorged females, L: larvae, SB: splenectomized calf.

A schematic representation of the experimental design used in this study is shown in Figure 1. The study was conducted in a herd of 26 3-year-old Brangus breed male bovines with high tick infestation from an enzootic area for *A. marginale* [11,30] in Gdor. Virasoro, Corrientes province, Argentina (28°03′00″ S 56°02′00″ W). Blood samples (10 mL) were collected from the jugular vein of all the animals and preserved in 3.8% sodium citrate for further analysis. Engorged female ticks (n = 10 per animal) were collected from three randomly selected animals and conserved alive in 5 mL plastic tubes with perforations in the lid to allow respiration.

Since *A. marginale* infection was confirmed by PCR (see following sections) in blood samples from all the bovines, including the three animals selected for tick collection, only one bovine (named donor calf) and its ticks (named EF 1–10) were randomly chosen to continue with this study.

Ticks were identified as *R. microplus* under a stereoscopic magnifier (10X–40X), according to taxonomic keys [31]. Six of the 10 female ticks (EF5 to EF10) were dissected (see details in the following section) to determine the presence of *A. marginale* in the salivary glands and ovaries. The other four female ticks (EF1 to EF4) were kept alive inside the plastic tubes for hemolymph sampling (see next section) and allowed to oviposit in an incubator at 28.0 °C and 80% relative humidity. Between days 25 and 32 after oviposition, larvae began to hatch. After identifying *A. marginale* in the engorged females E1 to E4 (hemolymph and/or bodies after oviposition) and in a sample of the larval pools laid by those ticks (named L1 to L4), we used the remaining live larvae fraction for an experimental infestation of a naïve splenectomized calf (SB) under controlled conditions. The animal used for the tick infestation experiment was an 18-month-old Hereford healthy steer, negative for Bovine viral diarrhea virus, Bovine rhinotracheitis virus (VHB-1), Bovine leukemia virus, Bluetongue virus, foot and mouth disease virus, and *Brucella* sp. as well as *A. marginale* infection according to cELISA and PCR analyses. In addition, five months before the infestation experiment, the animal had been vaccinated against *Babesia bovis* and *Babesia bigemina* using the attenuated M1A strains produced by EEA-Mercedes, INTA, Argentina (https://inta.gob.ar/servicios/vacuna-babesan, accessed on 28 June 2023) [32]. The corral used for the infestation experiment was especially refurbished to handle cattle and avoid the access of other vectors: cement floor and roof, walls of metallic mesh, and surrounded by a gutter for weekly pouring ASPERSIN^®^ (Biogénesis Bagó, Buenos Aires, Argentina).

A total of 320 mg of *A. marginale*-infected larvae (equivalent to approximately 16,000 individual larva [33]) were equally distributed inside four cloth bags of approximately 12 cm in diameter, each glued to the back of the animal on both sides of the spine. The skin had been shaved before gluing the bags. The bags were opened regularly to assess larvae development up to the engorged adult stage. To assess infection and the eventual development of the disease, the splenectomized calf was clinically monitored weekly by measuring rectal temperature and Packed Cell Volume (PCV) over a period of 35 days. The presence of *A. marginale* was also evaluated by microscopic observation of Giemsa-stained thin blood smears. Additionally, the blood samples collected in each monitoring instance were used for molecular analyses for the detection of *A. marginale* using PCR targeting *msp5* and *msp1β* genes, as described below.

The genotypic diversity of *A. marginale* was evaluated as described below, in the initial donor calf, the bodies of EF1–EF4 ticks after oviposition, the hatched larvae L1–L4, and the splenectomized calf infested with L1–L4 larvae.

### 2.3. Tick Dissection and Hemolymph Collection

Before dissection, ticks were washed with 1X phosphate buffered saline (PBS), then with 70% ethanol, and finally rinsed in bi-distilled water. The organ dissection was performed in Petri dishes containing 3 mL of sterile 1X PBS. An incision was made in the anterior part of the body to release the head and remove the organs using sterile forceps and needles [34,35]. The ovaries and salivary glands were identified and extracted, washed with 70% ethanol, rinsed in sterile 1X PBS, and placed individually in 0.2 mL tubes containing 10 µL of sterile 1X PBS.

For hemolymph collection from the ticks kept alive for oviposition (EF1–EF4), a leg was cut at the distal joint using small scissors, and hemolymph was collected with an automatic pipette tip and placed in a 0.2 mL tube containing cell lysis buffer. The hemolymph samples were used directly for PCR reactions, without previous DNA extraction.

### 2.4. DNA Extraction from Different Samples

DNA was extracted from blood samples, tick bodies after oviposition, tick organs, and larval pools (1 pool of ≈ 200 larvae per female). In the case of bovine blood samples, DNA was extracted from 400 μL of blood using the ADN PuriPrep-S kit (INBIO Highway, Buenos Aires, Argentina) according to the manufacturer’s instructions. The remaining samples were subjected to DNA extraction using the phenol/chloroform method and ethanol precipitation [36]. Prior to DNA extraction, tick bodies after oviposition and larvae were washed three times with 70% ethanol and rinsed in bi-distilled water [37], and then the specimens were crushed in liquid nitrogen. In the case of the tick organs, an overnight incubation with a cell lysis buffer containing Proteinase K (100 µg/mL) was performed before DNA extraction.

DNA quality and concentration were determined using a micro-volume spectrophotometer (NanoDrop ND-1000, Thermo Fisher Scientific, Waltham, MA, USA). All samples were stored at −20.0 °C until further use.

### 2.5. Anaplasma marginale Detection

*Anaplasma marginale* identification was carried out by amplifying two species-specific genes: *msp5*, a single-copy gene that encodes the outer major surface protein MSP5 [38]; and *msp1β*, a three-copy gene fragment that encodes the outer major surface protein MSP1b [39]. The molecular amplifications were performed in a 20 µL reaction mixture containing 0.4 μM of each primer, 0.2 mM of each deoxyribonucleotide triphosphate (INBIO Highway, Buenos Aires, Argentina), 0.5 U of Top Taq DNA polymerase (Qiagen, Hilden, Germany), 2 μL of 10X PCR buffer and ~100 ng of genomic DNA under published conditions [38,39]. Positive (DNA from *A. marginale* Mercedes strain) and negative (pure water) controls were included in the assay. Each amplified product (10 μL) was analyzed by electrophoresis in 1.5% agarose gel stained with ethidium bromide and a molecular size marker (1 Kb Plus DNA Ladder, Invitrogen, Carlsbad, CA, USA).

### 2.6. Amplification, Cloning, and Sequencing of the msp1α Gene

The *A. marginale* genotypes were assessed by amplifying and sequencing a fragment of the *msp1α* gene [40]. This gene is frequently used as a genetic marker in epidemiological studies since it encodes MSP1a protein, which varies in size among isolates due to different numbers of tandemly repeated 28–29 amino acid peptides.

The reaction was performed in 50 μL (0.4 µM of each primer; 0.2 mM of each deoxyribonucleotide triphosphate; 1.25 U of TopTaq DNA polymerase Qiagen, Hilden, Germany; 5 µL of 10× PCR buffer; and purified water to reach a final volume of 50 µL) using ~200 ng of genomic DNA. The amplified products were purified using a commercial kit (QIAquick PCR Purification Kit, Qiagen) according to the manufacturer’s instructions.

The amplified and purified PCR fragments were cloned into the pGEM-T easy^®^ vector (Promega, Madison, WI, USA) following the manufacturer’s instructions and transformed into DH5α *Escherichia coli* competent cells (prepared in-house) and selected on LB/ampicillin/IPTG/X-Gal plates. Recombinant plasmids from white colonies were purified using Wizard^®^ Plus SV Minipreps DNA Purification System (Promega) and sequenced using the universal primers T7 and SP6 with a Big Dye Terminator v3.1 kit and analyzed on an ABI 3130XL genetic analyzer (Applied Biosystems, Woburn, MA, USA), at the Genomic Unit (IABIMO, INTA-CONICET, Buenos Aires, Argentina). Both strands of the plasmid were sequenced to achieve greater reliability in the studied region. The total analyzed samples included 32 clones of the donor calf, 12 of EF, 8 of L, and 5 of the splenectomized calf. The complementary nucleotide sequences of each fragment were assembled and translated into the MSP1a protein sequence using the Vector NTI Advanced 10 program (Invitrogen, Waltham, MA, USA). Tandem repeats were manually identified in the amino acid sequences using the updated database from the RepeatAnalyzer software [41]. The eight novel repeats identified in the present study were named AR1 to AR8. Sequence variants were submitted to GenBank (accession numbers ON863929-ON863963) and are publicly available (Supplementary Figure S1 and Supplementary File S1).

## 3. Results

### 3.1. Natural Infection of Bovines and R. microplus Ticks with A. marginale

The presence of *A. marginale* in the 26 bovines from the enzootic region was confirmed by amplification of both *A. marginale msp5* and *msp1β* genes in each animal. *Anaplasma marginale* DNA was also detected in samples from tick organs (i.e., salivary glands and ovaries) dissected from five of the six *R. microplus* engorged females collected from the donor calf (Table 1 and Figure 1). From the selected set of *R. microplus* females allowed to oviposit, only one hemolymph sample tested positive according to the *msp1β* PCR analysis. Since the volume of the hemolymph samples was limited, we could only test them using this single target gene (*msp1β*). Larvae hatched from eggs laid by the four females EF1–EF4, as well as the spent females after oviposition, also tested positive for both *A. marginale* specific genes *msp5* and *msp1β*. Altogether, these results demonstrate pathogen acquisition by feeding female ticks, confirmed by bacteria presence in different body compartments of the vector as well as the transovarial passage of the pathogen from the engorged female to its offspring.

### 3.2. Experimental A. marginale Transmission from Newly Hatched Naturally Infected Larvae to Splenectomized Bovine

**Figure 2:**
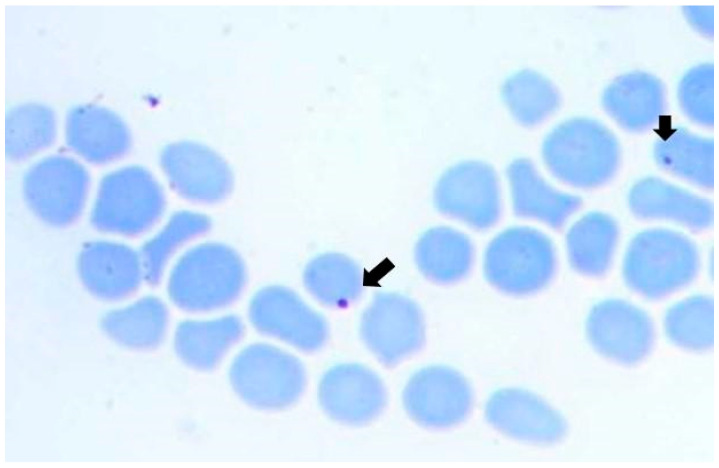
Blood smear from the splenectomized calf at day 22 post-infection and stained with May-Grunwald-Giemsa 1000X oil immersion. The arrows point out spherical inclusions suggestive of *A. marginale*.

Unfed larval offspring positive for *A. marginale*, laid by females collected from the naturally infected bovine, were used in an infestation experiment to test for *A. marginale* transmission. During the monitoring period, intracellular corpuscles suggestive of *A. marginale* were observed in a blood smear at day 22 post-infestation (Figure 2). Positive reactions for both target genes (*msp1β* and *msp5*) in a blood sample from day 22 confirmed the presence of *A. marginale*. On days 0 and 22 of the experiment, the PVC and rectal temperature of the splenectomized were 28% and 38.5 °C; and 27% and 39.0 °C, respectively. The experimentally infected bovine did not require an *A. marginale*-specific treatment since its clinical parameters remained stable within the reference range for bovine PCV and rectal temperature. Between days 28 and 35, post-infestation, approximately 200 larvae became engorged females.

### 3.3. Anaplasma Marginale Strain Genotyping during the Transmission Cycle

**Figure 3:**
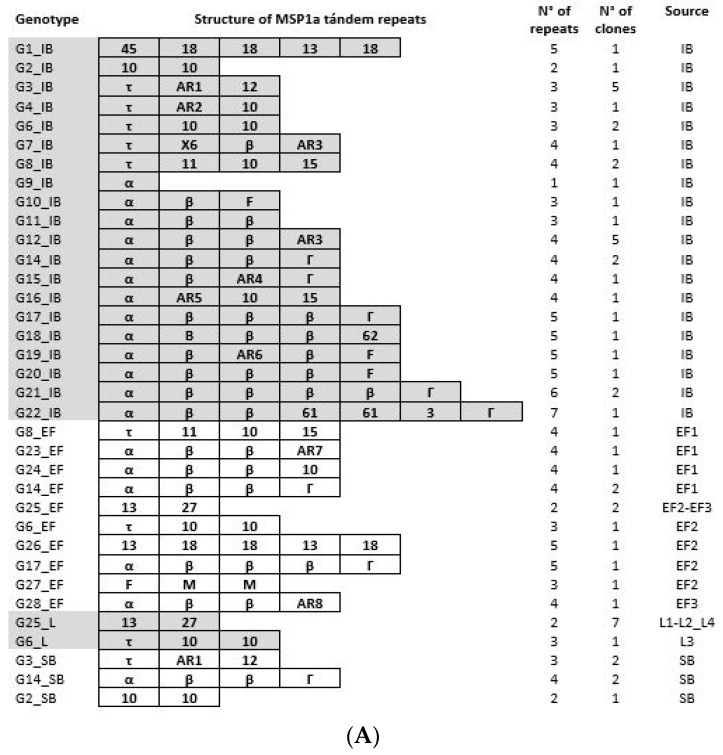

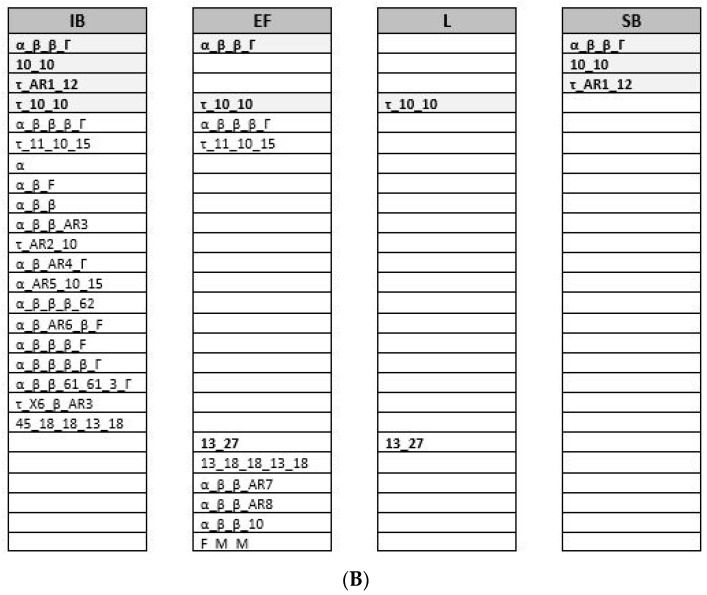
(**A**) *Anaplasma marginale* MSP1a tandem repeats for genotypes identified in the present study. The number of repeats that result in each genotype (N° of repeats), the frequency of genotype detection (N° of clones), and the source of the sample for each genotype are specified. (**B**) *Anaplasma marginale* MSP1a genotypes found in the different stages involved in the assay. IB: naturally infected bovine (donor calf), EF: engorged females, L: larvae, SB: splenectomized calf. Each cell contains a genotype. Genotypes considered to have been transmitted to larvae are highlighted and bold.

We performed *msp1α* genotyping to track the *A. marginale* strains involved in natural *A. marginale* infection of the selected bovine from the enzootic area as well as the genetic diversity during the pathogen transmission cycle from female ticks to larvae and subsequently to the splenectomized bovine. Different strains were identified across the stages of the transmission chain (Figure 3) and sequences obtained for the *msp1α* gene in this study varied between 346 and 1057 nucleotides (Supplementary File S1).

Natural infection of the donor calf involved multiple strains of *A. marginale*. A total of 20 genotypes were detected in this bovine, consisting of a combination of 21 Msp1a repeats, with a minimum of 1 (α) and a maximum of 7 (α-β-β-61-61-3-Γ) repeats (Figure 3). Eight of the Msp1a repeats identified here were not previously reported, namely AR1 to AR8 (Figure S1). Three of 4 engorged females contained 10 *A. marginale* genotypes and 6 of these genotypes were different from those found in the donor calf. The genotypes found both in donor calf and EF ticks (26 genotypes in all) may be potentially transmissible.

Genotypes found in the unfed larvae are representative of the transovarial transmission process of *A. marginale* from the females to their progeny, while genotypes found in the splenectomized calf are evidence of vector competence.

The most abundant genotypes identified in the donor calf were α-β-β-AR3 and τ-AR1-12 (5 clones of the 32 screened). Notably, the strain τ-AR1-12 (2 clones out of the 5) was also found in samples of the splenectomized calf after tick infestation. The splenectomized calf also contained the genotype 10-10 (1 clone out of 5). Additionally, α-β-β-Γ was present not only in the donor calf but also in EF (2 clones out of 12) and splenectomized calf (2 clones out of 5).

The genotype τ-10-10 occurred in the donor calf, EF, and larvae (2 clones out of 32 and 1 clone out of 12 and 8, respectively). Finally, genotype 13-27 was present both in EF (2 clones out of 12) and larvae (7 clones out of 8).

## 4. Discussion

In the present study, we provide strong evidence supporting the transovarial transmission of *A. marginale* in *R. microplus* ticks under natural conditions since *A. marginale* DNA was present in all the transmission-involved stages. The bacterium was present in naturally infected bovines, in different organs of engorged *R. microplus* females parasitizing the bovines, and in the larvae obtained from these female ticks. After using the larvae for infesting a splenectomized calf, we corroborated *A. marginale* infection both by DNA detection and in blood smears, which presented structures compatible with *A. marginale* infection. This finding supports the vector competence of *R. microplus* for *A. marginale* transmission. In this sense, recent studies reported evidence of transovarial transmission of other species in the genus *Anaplasma* by *Ixodidae* ticks as is the case for *A. platys* and *A. phagocytophilum*. For *A. platys*, a highly efficient transovarial transmission by *R. sanguineus* sensu stricto ticks has been documented [26], even though a previous study reported opposing results under different epidemiological conditions [42]. Also, for *A. phagocytophilum*, no transovarial transmission by *Ixodidae* ticks has been initially reported, except for *Dermacentor albipictus* [43]. In subsequent studies, *A. phagocytophilum* DNA has been detected in questing larvae of different tick species suggesting the possibility for transovarial transmission of the pathogen in those vectors [27,28,29]. In one of those studies [29], the authors inferred transovarial transmission under field conditions and also speculated that coinfection of the mother tick with other tick-borne microorganisms (*Borrelia* spp. and *Rickettsia* spp.) promotes transovarial transmission efficiency. Regarding our research and considering that the engorged females came from a region with high rates of coinfection with *Babesia bovis* and *B. bigemina* [44], undoubtedly transmitted by *R. microplus*, we could hypothesize that the transovarial transmission of *A. marginale* in pools of larvae could be linked to coinfection with protozoa. Ongoing studies from our group attempt to test this hypothesis.

The presence of *A. marginale* in ovaries is a necessary precondition for transmission to the offspring. Here, we detected *A. marginale* DNA using two different target regions both in salivary glands and ovaries, thus reinforcing that the transmission of *A. marginale* requires efficient invasion and replication of the bacterium in the tick tissues. Moreover, we detected *A. marginale* in hemolymph obtained from EF, leading to broad infection including organs such as the ovaries. Similarly, our study group has detected *A. marginale* in the ovaries of the three-host tick *Amblyomma sculptum* collected on giant anteaters. These results reinforce the evidence that, after being acquired in a blood meal, *A. marginale* could replicate and migrate to the ovaries of *Ixodidae ticks* [45]. Furthermore, the fact that we found six genotypes in the EF that were not present in the donor calf from which they were feeding can be explained by the transovarial acquisition from their female parental tick and not during the blood meal. On the other hand, it is important to consider that these six genotypes may have been present in the donor calf at relatively low abundance so that they were not captured in the cloning and sequencing process.

Previous studies evaluating the transovarial transmission of *A. marginale* in the monoxenic tick *R. microplus* concluded that this phenomenon did not occur under certain experimental conditions [20,21,22,23]. Given that many factors are involved in this type of process, the experimental conditions used in those studies may have failed to combine enough variables to reproduce such a complex phenomenon. In this regard, Shimada et al., (2004) [18] have shown that the offspring larvae of *R. microplus* females infected with *A. marginale* were positive through the use of PCR after being incubated at 18.0 °C, highlighting temperature as the critical environmental factor for the migration of *A. marginale* from the midgut to the *R. microplus* ovaries. The authors, however, have not evaluated whether these larvae were able to transmit this bacterium to calves. In a later work, Esteves et al., (2015) [23] demonstrated that a low temperature exerted negative effects on female fertility and egg development but had no influence on *A. marginale* transmission to the progeny. The authors attributed differences between the results from both studies, in part, to the variation between the strains used.

Here, we demonstrated vector competence after infesting a splenectomized calf using larvae coming from engorged females incubated at 28.0 °C, both by microscopy and by PCR assays targeting two separate specific gene markers. The fact that the splenectomized calf challenged by infected larvae showed no clinical signs could be due to the concurrence of many factors, such as the genetic background of the host, the bacterium load, and the care and feeding regime during the experimental procedure. Estrada et al., (2020) [46] have obtained a similar result by infesting cattle with *A. marginale*-positive *R. microplus* larvae. In this last case, larvae were obtained from engorged ticks incubated at 28.0 °C, that is, under equivalent conditions to those used in this work.

It has been extensively described that *A. marginale* strains differ in their infectivity to ticks [47,48] and in the extent to which they are transmissible by them [12]. The ability to infect the vector seems to depend on the surface adhesins MSP1a of *A. marginale* [40,49]. In the present study, after identifying *A. marginale*, we further characterized the *msp1α* genotypes involved throughout the infection process, thus providing key information on the transmission dynamics. By means of cloning and sequencing all *msp1α* fragments, we were able to identify 20 different strains in the donor calf among the 32 screened clones, which emphasizes the importance of this approach for describing the whole range of variants, including those with low abundance [50,51]. In spite of more than six genotypes per sample having been previously reported in highly prevalent regions [50,52], to the best of our knowledge, this is the first report of such a high number of genotypes within one bovine.

The accumulation of multiple strains of *A. marginale* in a host can be due to coinfection and/or superinfection, leading to what is recognized as complex infections [53]. This kind of process is commonly associated with high *A. marginale* prevalence combined with heavy tick burdens. The acquisition of two or more variants before (coinfection) or after (superinfection) the development of an adaptive immune response is difficult to distinguish in the case of the naturally infected bovines in our study since these animals inhabit an endemic (persistently infected) area. Nevertheless, as the experimentally infected splenectomized calf was originally *A. marginale* free, the three genotypes identified within this animal after being challenged by tick larvae infestation can be attributable to a coinfection event.

The occurrence of shared genotypes in the donor calf, EF, L, and splenectomized calf supports the circulation of *A. marginale* through all the different stages involved in the transmission cycle. Notably, the reduction in the number of unique genotypes along the transmission process reveals the bottleneck effect exerted by the tick vector (Figure 3). Similarly, under laboratory conditions, the genotype diversity of the tick-transmitted bacterium *Francisella novicida* was markedly reduced in the tick in relation to the mammalian host and this event depended on selective forces and stochastic factors [54].

Although researchers have demonstrated the competition between *A. marginale* genotypes during infection of the tick vector [55], in the case of *Borrelia burgdorferi*, facilitative interactions among genotypes in mixed infection may represent an advantage for the bacteria to establish infection in ticks [56]. Therefore, we should not exclude that an equivalent interaction could be the reason that leads to the transovarial transmission of *A. marginale* in highly prevalent regions. The identification of two equal genotypes (13-27 and τ-10-10) in EF and their offspring larvae, suggests transovarial transmission of *A. marginale* in *R. microplus*. This result supports that *A. marginale* can reach the ovaries of the engorged female, thus generating offspring larvae that harbor and are able to transmit the bacterium.

By using sequence similarity networks, Catanese et al., (2018) [57] have identified a group of seven Msp1a repeats that were considered to be central in the graph patterns by comparing its sequence structure with other Msp1a repeats across a great number of countries. They also seem to be common repeats because they were widely dispersed geographically. The authors speculated that central/common Msp1a repeats could be ancestral types that are widely distributed and structurally central. Interestingly, repeats 13 and 27 are two of the seven sequences that are both central and common, thus suggestive of tick influence in shaping the population structure of *A. marginale msp1α* genotypes.

Although the genotypes found in the splenectomized calf were undetectable in larvae, *A. marginale* strains could only have been transmitted if transovarial transmission had occurred. In our experimental design, the larvae used for genotyping were not the same as those used for the experimental infestation. The fraction of the larvae (L1–L4) used for genotyping (around 200 individuals) represented approximately 4.5% of each of the four populations (320 mg on average in total) seeded on the splenectomized calf for the vectorial competence assay. Thus, the mismatch between the genotypes of L1–L4 and the splenectomized calf could be due to the low coverage of larvae genotype richness. Moreover, we may have missed some low-abundance strains while genotyping the donor calf and the splenectomized calf [51]. Similarly, the transovarial transmission of *Anaplasma platys* by *R. sanguineus* was demonstrated in spite of the fact the eggs from the first generation were negative for *A. platys* probably because of the low prevalence of pathogen infection [26].

Altogether, our results reveal a concealed aspect of the biological cycle of *A. marginale*. The broad range of variables involved in the pathogen-vector-host interactions may explain the apparent inconsistencies among experimental demonstrations of transovarial transmission in *Anaplasma* spp. [26]. Current studies approaching pathogen genotypic diversity within the host and the tick vector will contribute to a better understanding of tick-borne bacterial transmission.

## 5. Conclusions

In the present study, we demonstrated the transovarial transmission of *A. marginale* in *R. microplus* ticks under natural conditions and we corroborated the vector competence of the infected *R. microplus* larvae to transmit *A. marginale* to a susceptible bovine in an experimental infection assay. Furthermore, we observed that *A. marginale* strains experience bottlenecks during the transmission cycle, from a naturally infected bovine to the tick vector and its offspring, and then to a susceptible bovine. *Anaplasma marginale* genotyping provides key information for understanding transmission dynamics and further studies should test whether some strains have a selective advantage over others during tick transovarial transmission and whether by becoming dominant, a strain could cause outbreaks leading to major economic losses.

## Figures and Tables

**Table 1:** Results for the molecular amplification of the three target genes used in the study (*msp1β*, *msp5*, and *msp1α*) for the different samples tested from the diverse hosts involved in the *A. marginale* transmission cycle (IB: donor calf, EF, Larvae, and SB: splenectomized calf). ND: not determined, NA: not amplified.

Sample	*msp1β*	*msp5*	*msp1α*
**IB**	**POSITIVE**	**POSITIVE**	**POSITIVE**
**EF**			
*Hemolymph*			
EF 1	**POSITIVE**	ND	ND
EF 2	NEGATIVE	ND	ND
EF 3	NEGATIVE	ND	ND
EF 4	NEGATIVE	ND	ND
*Bodies after oviposition*			
EF 1	**POSITIVE**	**POSITIVE**	**POSITIVE**
EF 2	**POSITIVE**	**POSITIVE**	**POSITIVE**
EF 3	**POSITIVE**	**POSITIVE**	**POSITIVE**
EF 4	**POSITIVE**	**POSITIVE**	NA
*Salivary glands*			
EF 5	**POSITIVE**	**POSITIVE**	ND
EF 6	**POSITIVE**	**POSITIVE**	ND
EF 7	**POSITIVE**	**POSITIVE**	ND
EF 8	**POSITIVE**	**POSITIVE**	ND
EF 9	**POSITIVE**	**POSITIVE**	ND
EF 10	NEGATIVE	NEGATIVE	ND
*Ovaries*			
EF 5	**POSITIVE**	**POSITIVE**	ND
EF 6	**POSITIVE**	**POSITIVE**	ND
EF 7	**POSITIVE**	**POSITIVE**	ND
EF 8	**POSITIVE**	**POSITIVE**	ND
EF 9	**POSITIVE**	**POSITIVE**	ND
EF 10	NEGATIVE	NEGATIVE	ND
**Larvae**	**POSITIVE**	**POSITIVE**	**POSITIVE**
L1	**POSITIVE**	**POSITIVE**	**POSITIVE**
L2	**POSITIVE**	**POSITIVE**	**POSITIVE**
L3	**POSITIVE**	**POSITIVE**	**POSITIVE**
L4	**POSITIVE**	**POSITIVE**	**POSITIVE**
**SB**	**POSITIVE**	**POSITIVE**	**POSITIVE**

## Data Availability

All data generated or analyzed during this study are included in this published article. The datasets associated with *A. marginale* genotypes generated were submitted to the GenBank database at https://www.ncbi.nlm.nih.gov/gene (accessed on 28 June 2023) and received the following accession numbers: ON863929-ON863963.

## Supplementary Materials

The following supporting information can be downloaded at: https://www.mdpi.com/article/10.3390/pathogens12081010/s1. The *msp1α* nucleotide and MSP1a amino acid sequences associated with each genotype and sample can be found in Supplementary File S1. Figure S1. A: Encoded sequence for repeat A is used as reference. AR1–AR 8: repeats reported for the first time in the present study.
