# Supplementary figures and images for: Transovarial Transmission of Anaplasma marginale in Rhipicephalus (Boophilus) microplus Ticks Results in a Bottleneck for Strain Diversity
Source: Pathogens. 2023 Aug 2;12(8):1010. doi: 10.3390/pathogens12081010 (PMC10459439; doi:10.3390/pathogens12081010)

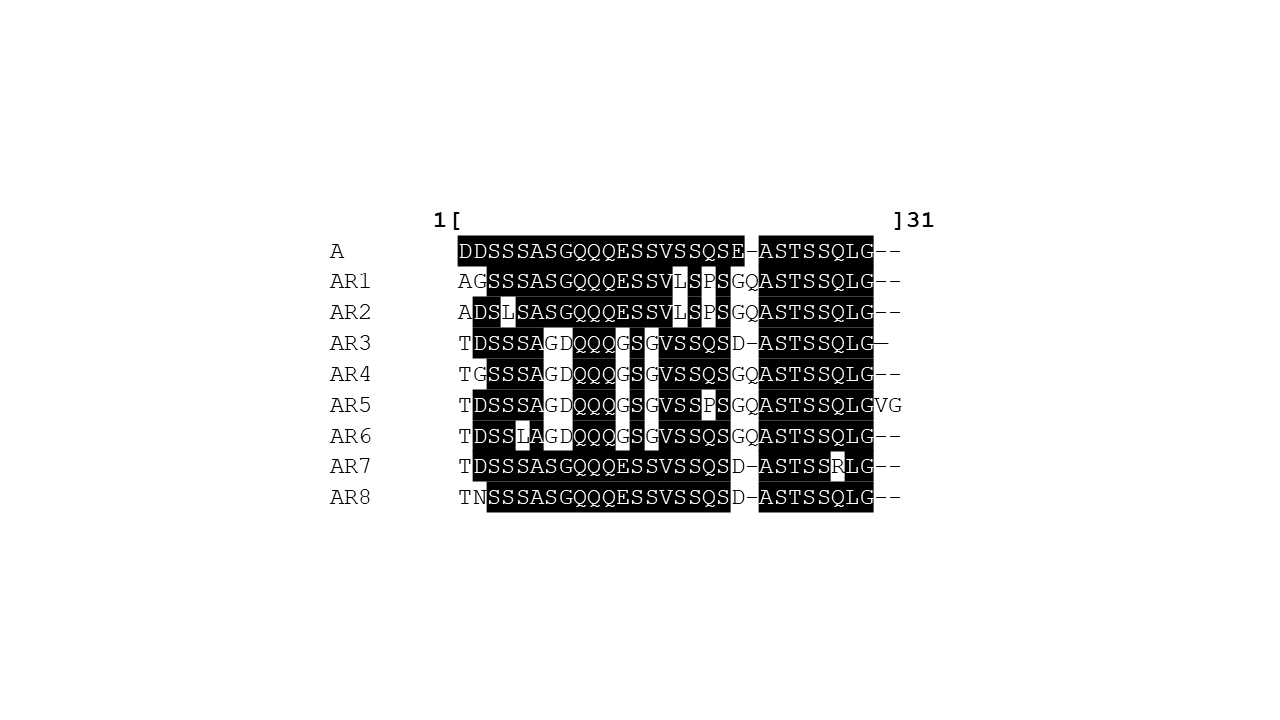

Supplement: Supplementary file 1 [file pathogens-12-01010-s001.zip › pathogens-2505086-supplementary.TIF]
